# Therapeutic Potential of Microalgae-Derived Bioactive Metabolites Is Influenced by Different Large-Scale Culture Strategies

**DOI:** 10.3390/md20100627

**Published:** 2022-09-30

**Authors:** Jezabel Garcia-Parra, Claudio Fuentes-Grünewald, Deyarina Gonzalez

**Affiliations:** 1 Faculty of Medicine, Health and Life Science, Swansea University, Singleton Park, Swansea SA2 8PP, UK; 2 Faculty of Science and Engineering, Swansea University, Singleton Park, Swansea SA2 8PP, UK; 3 Beacon Development, King Abdullah University of Science and Technology, Thuwal 23955-6900, Saudi Arabia

**Keywords:** microalgae, cancer cells, antitumour, biomass, therapeutic, biotechnology

## Abstract

Microalgae have been identified as one of the most promising sources of novel bioactive compounds for biomedical applications, the food industry, and cosmetics. In the last decade, several biotechnological developments have facilitated the identification of a growing number of compounds as well as the study of optimal microalgae culture conditions for the production of biomass enriched in specific molecules of interest. In this study, two common commercial marine microalgae (*Nannochloropsis oculata* and *Porphyridium purpureum*) were cultured in standard and nutrient-stressed conditions and the obtained biomass extracts were assessed for their potential to inhibit cancer cell proliferation and migration as well as their antioxidant activity. Results from viability in 2D and 3D cancer cell models showed an enhancement of the antitumour activity of *P. purpureum* in the 3D model compared to 2D, together with a greater capacity to reduce the migration capacity of cancer cells with the biomass from nutrient-stressed conditions, whereas the antioxidant activity of *N. oculata* decreased when exposed to nutrient-stressed conditions. To date, this is one of the few studies that proves that controlled changes in large-scale culturing conditions such as nutrient depletion have a relevant impact in the bioactivity of the biomass on cancer cells.

## 1. Introduction

Currently, one of the main research emphases in the biotechnology field is the isolation and characterisation of biological active compounds extracted from natural sources. To date, there are 20 approved drugs using compounds from marine origin for different treatment indications, more than 25 in clinical trials and hundreds of compounds reported to be in preclinical studies [1,2]. Most of the marine-derived compounds in the clinical settings have been initially obtained from wild sponges, tunicates, and molluscs/cyanobacteria, but the production of therapeutic compounds from these organisms is not sustainable or suitable. Comparably, the use of microalgae-derived compounds is still in the early days of exploration for drug development. 

Microalgae are an extremely diverse group of unicellular photosynthetic microorganisms that can usually be found in fresh-water and marine systems. Their diversity and the ability to adapt and survive in different environments makes them a vast source of bioactive molecules. For this reason, microalgae biomass production is one of the fastest growing biotechnology fields in recent years [3]. This increased knowledge of microalgal biomass production in different culture-controlled systems is supported by the boost in the industrial use of the metabolites obtained from microalgae, such as: essential fatty acids like eicosapentaenoic acid (EPA), docosahexaenoic acid (DHA), arachidonic acid (AA), pigments (carotenoids, phycobiliproteins), proteins, exopolysaccharides (EPS), and macrolides, among others [3]. All these metabolites extracted from microalgae biomass exhibit different applications, ranging from human and animal nutrition, pharmaceutical, cosmetic, and wound healing, among others [4]. For instance, drugs derived from natural products have an enormous impact on the development of targeted therapies with superior clinical efficacy and decreased toxicity, and this is exemplified by auristatins, synthetic products that are structurally related to dolastatin 10. Auristatins, highly cytotoxic drugs, are the most used payloads in antibody–drug conjugates (ADCs), including Food and Drug Administration (FDA)-approved Adcetris^®^, Polivy^®^, Padcev^®^, Blenrep, and Tivdak^®^ [5].

Currently, microalgae are one of the most studied natural raw materials in different industrial applications and they have been identified as one of the most promising sources of bioactive compounds for biomedical applications [3,4,6]. 

Specific examples of groups of molecules from microalgae that are currently in preclinical studies for therapeutic applications include toxins and polyketides from dinoflagellates, such as amphidinolides and colopsinols with potent antitumour properties, protoceratins and pectenotoxins, also candidates for chemotherapeutic agents, and ceramides with antileukemic activity, among many other potent bioactive compounds [7]. Further identified compounds with anticancer activity described in other species of microalgae also include: specific polyunsaturated aldehydes (PUAs) from marine diatoms (*Thalassiosira rotula, S. costatum,* and *P. delicatissima*) that have shown great antiproliferative activity in cancer cells [8], fucoxanthin, which has been described to possess the greatest anticancer activity, compared to other carotenoids, against tested prostate cancer cell lines [9], or stigmasterol (from *Navicula incerta*), whose phytosterol-like structures have been shown to induce apoptosis in human liver cancer cells [10]. Noteworthy, polysaccharides, especially exopolysaccharides, can be used for a wide range of biomedical applications, including anti-inflammatory, immunomodulatory, anti-glycaemic, antitumour, antioxidant, anticoagulant, antilipidemic, antiviral, antibacterial, and antifungal activities (reviewed in [11]). *Porphyridium* sp., *Chlorella* sp., and *Arthospira* sp. have been studied to produce these active compounds. Hence, a growing number of microalgae species are being screened for their biological activities, including anticancer activity, and their molecule composition profiles [12,13]. Additionally, an increasing number of studies have shown changes in macromolecule composition upon changes in abiotic parameters [14,15,16,17], but studies comparing different culture conditions, macromolecule composition, and bioactivity on cancer cells in the same study are less common [18,19]. It must be noted that most of the microalgae studies for biological studies were performed at the lab scale (few litres of culture) and not in real environments in large-scale microalgae facilities, as in this study. The growing number of bioactive molecules reported in microalgae will soon demand parallel biotechnological studies to describe the optimal culture and harvesting approaches to achieve enrichment in the bioactive metabolites of interest.

Cancer, as one of the leading causes of death worldwide, remains one of the most studied biomedical applications of natural products [20]. In females, ovarian cancer (OC) is considered the deadliest among gynaecological cancers, with a poor prognosis. Despite advances in anticancer therapies in recent years, novel efficacious therapeutics and targeted delivery vehicle strategies are vital to improve ovarian cancer survival rates. In general, first-line cancer treatment options include tumour-reductive surgery, radiotherapy, platinum/taxane-based chemotherapies, and targeted therapies. However, the majority of ovarian cancer patients receiving chemotherapy are prone to chemotherapy resistance, and hence the development of novel compounds to use as targeted therapies remains the main challenge in ovarian cancer treatment [21]. 

As a proof of concept, in this study we have used SKOV3, a cell line traditionally used as a model of an aggressive OC subtype to identify microalgae biomass with biomedical applications [22]. We obtained biomass from two common commercial marine microalgae strains (*Nannochloropsis oculata* and *Porphyridium purpureum*) grown at a large scale (>800 L) under standard and stressed (nutrient-depleted) conditions. The extracted biomasses were evaluated for their antitumour activities using different cell culture models, including 2D and 3D cultures, as well as their anti-cell migration properties using the 2D cell migration assay. To our knowledge, this is the first study using these microalgae strains that compares the antitumour activities of biomass obtained from different large-scale culture approaches.

## 2. Results

### 2.1. Microalgae Strain Performance in Photobioreactors in Standard and Stressed Conditions

**Figure 1:**
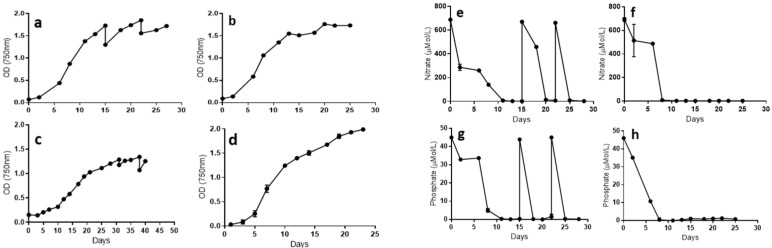
Growth curves and nutrient uptake in standard and nutrient-stressed culture conditions for *Nannochloropsis* and *Porphyridium*. (**a**) *Nannochloropsis* fed-batch culture mode, (**b**) *Nannochloropsis* batch culture mode, (**c**) *Porphyridium* fed-batch culture mode, and (**d**) *Porphyridium* batch culture mode. (**e**) Nitrate uptake in *Nannochloropsis* fed-batch culture mode, (**f**) nitrate uptake in *Nannochloropsis* batch culture mode, (**g**) nitrate uptake in *Porphyridium* fed-batch culture mode, and (**h**) nitrate uptake in *Porphyridium* batch culture mode.

**Figure 2:**
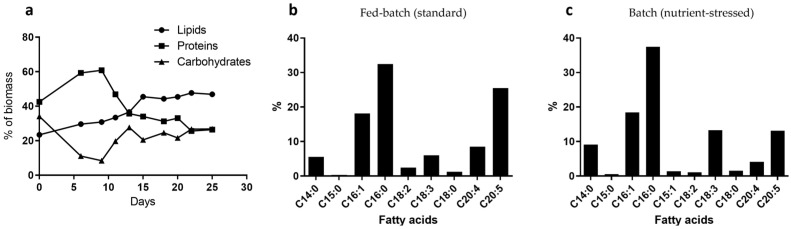
Comparison of macromolecule composition from standard and nutrient-stressed cultures of *Nannochloropsis*. (**a**) Macromolecules’ evolution during batch culture. (**b**) Fatty acid profile of the fed-batch (standard) condition. (**c**) Fatty acid profile of the batch (nutrient-stressed) condition.

In this study, two microalgae culture growth approaches (fed-batch and batch) were used to evaluate whether the different biomasses could show differences in the bioactivity in cancer cells. Fed-batch culture allows a stable and reliable biomass production, adding in a continuous basis nitrogen source, promoting a stable growth rate (Figure 1a,c,e,g). In contrast, the batch growth approach includes an initial addition of nutrients and bringing the biomass production to its maximum, and at the same time depleting all nutrients available, reaching the stationary phase where the microalgae is left in starvation to induce biomass macromolecule modification (Figure 1b,d,f,h). A similar growth approach (targeting high-value products) has been described before for other microalgae, such as *Nannochloropsis oculata,* or even for a totally different microalgae group such as dinoflagellates [23]. Figure 1 shows the two suggested culture approaches in *Nannochloropsis* (Figure 1a,b) and in *Porphyridium* (Figure 1c,d). *Nannochloropsis* has similar trends in terms of growth rate, *µ*: 0.34 day^−1^ (Figure 1a) for semicontinuous and *µ*: 0.36 day^−1^ in batch mode (Figure 1b), and comparable nutrient uptake, where nitrate uptake in batch culture was 3.5 µM/h (Figure 1h), similar to the fed-batch culture (3.2 µM/h, Figure 1g). From these cultures, yields (g/L) at harvest point (final day) were the following: *Nannochloropsis* fed-batch (standard): 1.50 g/L, *Nannochloropsis* batch (nutrient-stressed): 1.43 g/L, *Porphyridium* fed-batch (standard): 1.32 g/L, and *Porphyridium* batch (nutrient-stressed): 0.97 g/L. The biomass composition at harvest point in both approaches has differences in terms of macromolecule composition, especially in the lipids fraction for the *Nannochloropsis* strain (Figure 2b,c). For the *Porphyridium* strain, an analogous behaviour was noticed in terms of growth (Figure 1c,d) and the macromolecule composition at harvest point (please see previous published work from Fuentes-Grunewald et al. [14]). 

### 2.2. Macromolecule Composition in Microalgae Strains Is Influenced by Culture Conditions

The approximate macromolecule composition of *Nannochloropsis* biomass obtained from the fed-batch culture at day 15 of culture (standard, Figure 1a) accounted for a lipid content of 31.8%, protein content of 51.4%, and carbohydrate content of 16.7%. In the case of biomass obtained at the final harvest (day 25, Figure 2a) of batch culture (nutrient-stressed), the macromolecule composition accounted for a lipid content of 46.9%, protein content of 26.4%, and carbohydrate content of 26.8%. It is clear from the results obtained that nutrient depletion induces changes in the biomass macromolecule composition. The FTIR analysis allowed the real-time monitoring of the macromolecule composition trend in *Nannochloropsis* biomass as it grew in batch mode and was exposed to nutrient depletion during the exponential phase (Figure 2a) (see Supplementary Figure S1 for representative FTIR spectrums). A boost in the protein content was detected for the first eight days of culture (full nutrient conditions) and then a drop in proteins was observed once the nutrients (especially nitrate) were depleted. An initial carbohydrate increase, and subsequentially a lipid increase, was detected (day nine onwards). It is known that the carbohydrates (such as starch) and lipids (such as triacyl glycerides, TAG) act as energy storage in photosynthetic cells, and both used the same metabolic pathway for energy accumulation, which is clearly seen in Figure 2a. Another proxy used for macromolecules’ differentiation between standard and stressed microalgal biomass is the fatty acid profile. As shown in Figure 2b,c, when the *Nannochloropsis* biomass has full nutrient standard conditions, the amount of polyunsaturated fatty acids (PUFAs), and especially the eicosapentaenoic acid (C20:5*n*3), is higher (Figure 2b), accounting for nearly double the concentration when compared with stress conditions (Figure 2c). In contrast, the amount of TAGs, especially palmitic acid (C16:0), increases during nutrient-starvation compared with standard conditions.

**Figure 3:**
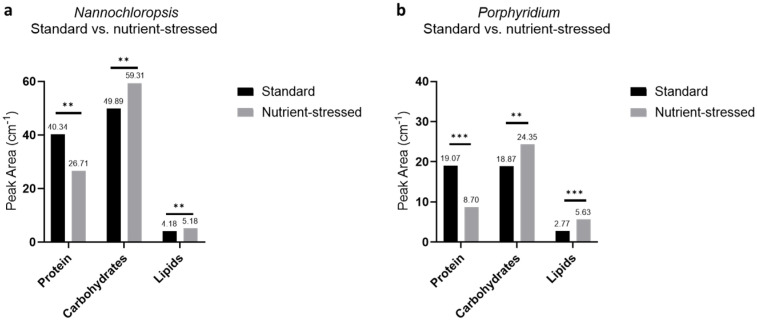
Comparison of macromolecule composition by FTIR assay from standard and nutrient-stressed cultures of *Nannochloropsis* and *Porphyridium*. Graphs from *Nannochloropsis* (**a**) and *Porphyridium* (**b**) show peak areas in cm^−1^ of overall protein, carbohydrate, and lipid contents obtained from the FTIR spectrum. Black and grey bars correspond to biomass from standard and nutrient-stressed conditions, respectively. To determine the lipid content, the methyl and methylene groups were found at 2850–2970 cm^−1^, carbohydrate content was found at 900–1200 cm^−1^, and protein content was determined using the Amide II peak at 1540–1545 cm^−1^. *T*-test analysis confirmed that there are significant differences between protein, carbohydrates, and lipids (** *p* ≤ 0.01, *** *p* ≤ 0.001).

Additionally, a comparison of the macromolecule composition of both target microalgae biomasses at the harvest point for each culture condition was carried out with the FTIR assay. The data highlight again the macromolecular changes induced by altering the culture conditions and the moment of harvesting the biomass. As observed in Figure 3a, biomass from *Nannochloropsis* grown under nutrient-stressed conditions, compared to standard conditions, displayed a significant reduction in the protein content (protein peak areas from standard and stressed: 40.36 and 26.71 cm^−1^, respectively, *p* = 0.0006), whereas carbohydrates and lipids exhibited an increase (carbohydrate peak areas from standard and stressed: 49.89 and 59.31 cm^−1^, respectively, *p* = 0.00135; lipid peak areas from standard and stressed: 4.18 and 5.18 cm^−1^, respectively, *p* = 0.0086). Similarly, *Porphyridium* also exhibited the same trends (Figure 3b). Biomass extracts from nutrient-stressed conditions, compared to standard conditions, displayed a significant reduction in the protein content (protein peak areas from standard and stressed: 19.07 and 8.07 cm^−1^, respectively, *p* < 0.001), whereas carbohydrates and lipids exhibited an increase (carbohydrate peak areas from standard and stressed: 18.87 and 24.35 cm^−1^, respectively, *p* < 0.005; lipid peak areas from standard and stressed: 2.77 and 5.63 cm^−1^, respectively, *p* < 0.001). 

### 2.3. Microalgae Biomass Extracts from Standard and Stressed Cultures Exhibit Antiproliferative Activity in Two- and Three-Dimensional OC Cell Culture Models

**Figure 4:**
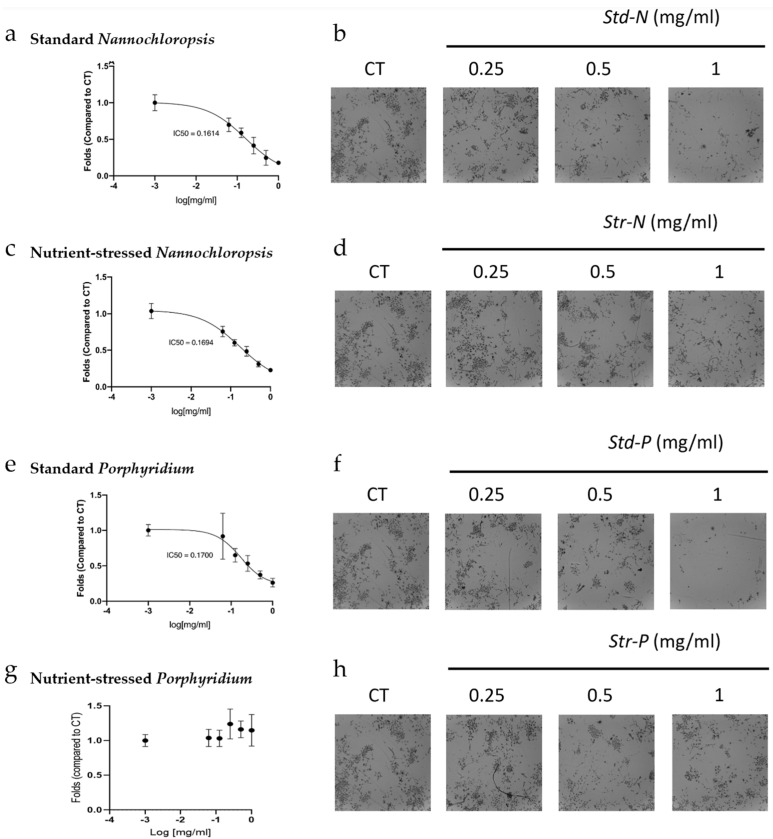
Antiproliferative effect of biomass extracts from *Nannochloropsis* and *Porphyridium* in 2D cultures of SKOV3 cells. Cells were plated and exposed to either the vehicle or different concentrations (0.0625, 0.125, 0.25, 0.5, and 1 mg/mL) of each biomass extract for 72 h. (**a**,**c**,**e**,**g**) Graphs from *std-N*, *str-N*, *std-P,* and *str-P,* respectively, showing fold change in cell viability compared to control. IC_50_ values were determined with GraphPad using a non-linear regression curve fit with a four-parameter model. (**b**,**d**,**f**,**h**) Picture panels from *std-N*, *str-N*, *std-P*, and *str-P* from crystal violet staining. Data are shown as means ± standard deviation, and experiments were performed in triplicates (*n* = 3).

Methanolic extracts obtained from the biomasses harvested from these different microalgae culture conditions were used to evaluate whether the differences observed in macromolecule composition could have an impact on the biological effects on cancer cell cultures. For this purpose, different types of cell culture models, including 2D and 3D models, were used in this study to evaluate the effects on viability upon treatment with microalgae biomass. Although 2D cancer cell models are the traditional cell culture models that use cells growing as a monolayer attached to the assay plate, 3D cancer cell models are becoming key to study properties of novel biomolecules as they are more complex culture models that use cells growing unattached, self-assembled in three-dimensional structures [24]. This allows a better mimicking of features described in the tumour environment by recapitulating some of the structure complexity, access to nutrients and oxygen, and penetration of drugs in the tumour mass. Three-dimensional cancer cell models can offer different levels of complexity, from containing a single type of cells to more elaborate mixtures of different types of cells and components of the extracellular matrix, to 3D structures differentiated from a single original stem cell (organoids), or actual pieces of tissue to directly assess the effects of drugs (ex vivo) [25,26,27]. As a first approach, the antiproliferative capacity of these four different biomass extracts was assessed in SKOV3, grown as two-dimensional monolayer cell cultures (2D cultures) using the RealTime-Glo™ MT Cell Viability assay. For this assay, cells were exposed to a range of biomass concentrations (from 0.0625 to 1 mg/mL) and the cell proliferation was monitored every 24 h for a total of 72 h. After 72 h, standard-*Nannochloropsis* (*std-N*), stressed-*Nannochloropsis* (*str-N*), and standard-*Porphyridium* (*std-P*) biomass extracts decreased cell proliferation in a dose-dependent manner (Figure 4a,c,e). The IC_50_ obtained at 72 h was 0.161 mg/mL for *std-N*, 0.169 mg/mL for *str-N,* and 0.17 for *std-P*. The biomass extract from stressed-*Porphyridium* (*str-P*) did not show any statistically significant decrease in cell proliferation in this 2D cell culture model (Figure 4g). As an alternative to the more sensitive luminescence detection of cell growth, changes in cell proliferation were also assessed by traditional crystal violet staining of 2D SKOV3 cell cultures exposed to the three highest treatments of biomass extracts for 72 h for qualitative confirmation purposes. The results obtained with this assay reproduced the same dose-dependent antiproliferative effect as observed before for *std-N*, *str-N,* and *std-P* (Figure 4b,d,f). *Str-P* did not modulate cancer cell proliferation (Figure 4h).

**Figure 5:**
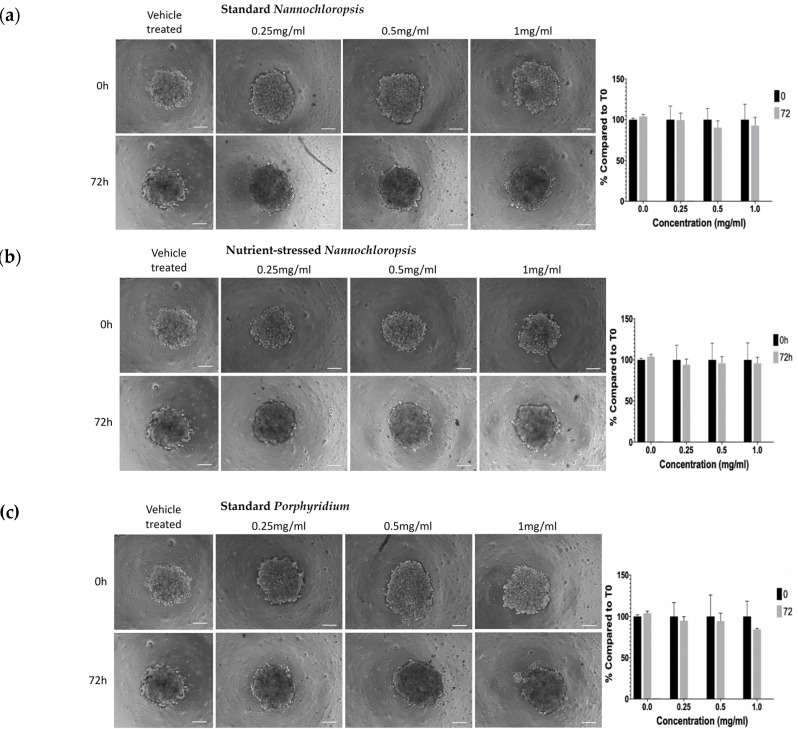

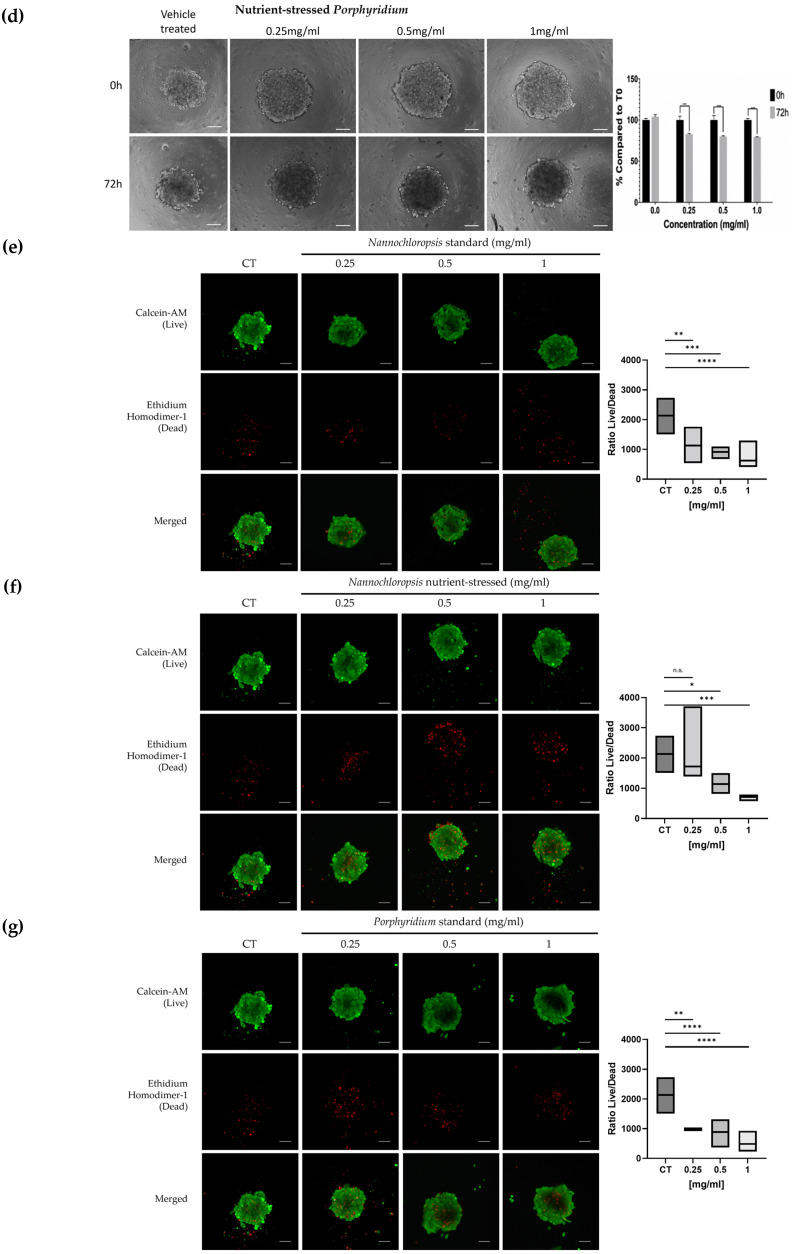

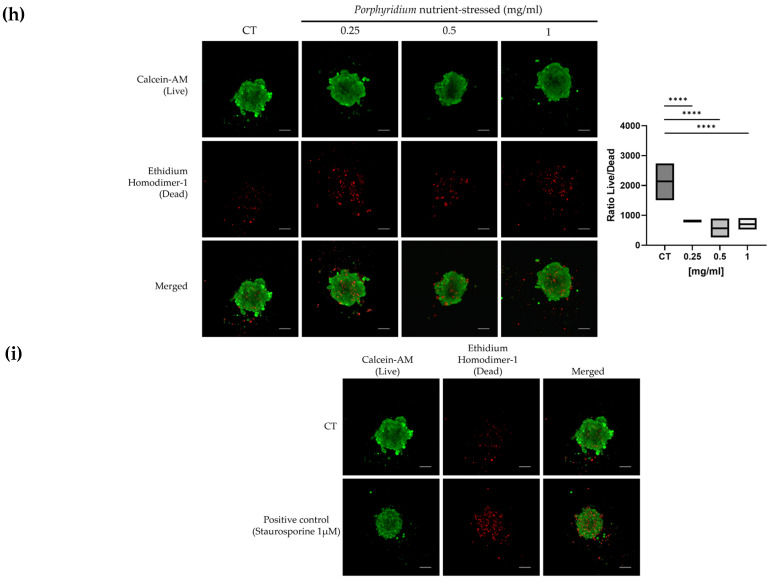
Effect of biomass extracts from *Nannochloropsis* and *Porphyridium* in 3D cultures of SKOV3 cells. For 3D culture models, SKOV3 cells were grown unattached in ULA plates and treated with 0.25, 0.5, or 1 mg/mL of biomass extract for 72 h. For size reduction effects: (**a**–**d**) graphs show spheroid size as percentage compared to respective size of each spheroid at time 0 h. Each graph has its associated representative 10× magnification images of spheroids at 0 and 72 h. Scale bar 50 µm. For the 3D live/dead assay: (**e**–**h**) graphs show the ratio of live/dead fluorescent intensity signal obtained from the image analysis of maximum-intensity projections from Z-stack of spheroids. Each graph shows its respective representative 10× magnification images of spheroids at 72 h. (**i**) Representative images of the positive control (Staurosporine 1 µM, 72 h). Green fluorescence is Calcein-AM (live signal), red fluorescence is the Ethidium Homodimer−1 (dead signal). Data are shown as means ± standard deviation, and experiments were performed in triplicates (*n* = 3). Scale bar 50 µm. One-way ANOVA test confirmed significant differences compared to the vehicle-treated condition. * *p* < 0.05, ** *p* < 0.01, *** *p* < 0.005, **** *p* < 0.001.

To further evaluate the antiproliferative activity of these biomass extracts, a three-dimensional (3D) culture spheroid model was used. Here, SKOV3 cells were grown in suspension in ultra-low attachment plates to induce their self-organisation into 3D spheroid structures. Three-dimensional cell cultures are in general less sensitive to some therapies due to several reasons, including reduced penetration of compounds into the core of the 3D structure and the proliferative status of cells in the spheroid, among others [28,29]. Expecting a reduced effect on viability compared to 2D, SKOV3 spheroids were exposed to the 3 highest concentrations of biomass extracts (0.25−1 mg/mL) for 72 h. Bright-field microscope pictures of each spheroid were taken at time 0 and time 72 h and the % of spheroid size reduction compared to its respective time, 0 h, was calculated by image analysis. As observed in Figure 5a–d, at 72 h of treatment, spheroids treated with vehicle showed a small increase in size compared to time 0 h, as expected, while spheroids treated with biomass extracts from *std-N*, *str-N,* and *std-P* showed a small decrease in size (not statistically significant). Interestingly, spheroids treated with *str-P* biomass extracts showed a 20% reduction in size compared to time 0 h (0.25 mg/mL, *p* ≤ 0.0001; 0.5 mg/mL, *p* ≤ 0.0001; 1 mg/mL, *p* ≤ 0.0001). 

In parallel, a live/dead fluorescent staining adapted to 3D cultures was used to study the viability of SKOV3 spheroids upon exposure to these four biomass extracts [26]. Using the same time and concentration conditions as described above, all the spheroids treated with the highest doses (1 and 0.5 mg/mL) showed a decrease in the intensity of green fluorescence (live signal) and an increase in the red fluorescence (dead signal) compared with control spheroids, which was expressed as the ratio of live versus dead signals (Figure 5e,h). In the case of this 3D live/dead assay, the significant decrease in these ratios upon treatment was similar between standard and stressed counterparts for both microalgae strains’ biomass extracts. 

### 2.4. Microalgae Extracts from Stressed Cultures Reduce the Migration Capacity of OC Cells 

**Figure 6:**
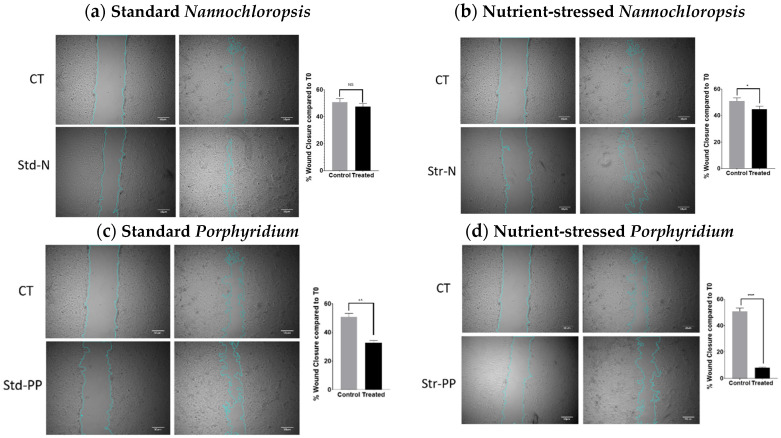
Effect of biomass extracts from *Nannochloropsis* and *Porphyridium* on migration. Effect on cell migration evaluated using the scratch wound-healing assay on SKOV3 monolayers treated with media containing 0.0625 mg/mL of standard *Nannochloropsis* (**a**), nutrient-stressed *Nannochloropsis* (**b**), standard *Porphyridium* (**c**), and nutrient-stressed *Porphyridium* (**d**) biomass extracts. A scratch was performed in the confluent monolayer and observed over 48 h. Left panels show representative microscopy images from 0 and 48 h at 4× magnification, where dotted lines define the margins of the wound area. Right graphs plot the quantification of migration as a percentage of wound closure compared to time 0 h (T0). Grey bars show the vehicle-treated group, and black bars show the biomass-treated group. The image analysis to quantify the wound closure was performed with ImageJ using the plugin “Wound-healing size tool”. *T*-test confirmed significant differences between control and treated groups (* *p* < 0.05, ** *p* < 0.005, *** *p* < 0.001).

Cell migration is one of the processes observed during metastasis of cancer cells. To study the capacity of *Nannochloropsis* and *Porphyridium* biomass extracts to alter the migration capacity of cancer cells, the wound-healing assay was used. For this purpose, a confluent 2D monolayer of SKOV3 cells was scratched, treated, and observed for 48 h. To make sure the reduction of migration was not due to cell death, a dose with no reduction of cell proliferation at 48 h (0.0625 mg/mL) was used. FBS-free media was used during this assay to reduce the cell proliferation, so it does not interfere with the measurement of cell migration. The percentage of wound closure was calculated comparing the same wound areas at time 0 and 48 h. As shown in Figure 6, wounds treated with vehicle displayed 50.9% wound closure at 48 h. When the wounds were treated with *std-N,* the wound closure was similar to the control (47.5%, n.s.), whereas when *str-N* biomass extract was used, the wound closure percentage was slightly decreased compared to the vehicle condition (44.8%, *p* < 0.0432). In the case of the biomass extract from *Porphyridium,* the results showed greater differences. Cells treated with the standard biomass extract had a more evident reduced capacity to migrate, showing 32.8% of wound closure (*p* < 0.005), while treatment with the stressed biomass extract enhanced this reduction in the migration capacity, exhibiting only 8.17% of wound closure (*p* < 0.001).

### 2.5. Microalgae Extraction Methods and Culture Strategy Influence the Antioxidant Activity of Microalgae Biomass Extracts

Finally, the potential antioxidant activity of these extracts was also evaluated to further demonstrate how differences in the culture conditions of microalgae can alter the composition of macromolecules, subsequently inducing changes in microalgae extracts’ properties. For this evaluation, the free radical scavenging activity (DPPH) method was used only to assess biomass extracts from *Nannochloropsis*. Methanolic extracts from biomass grown under standard culture conditions (*std-N*) showed a 50.31% inhibition of DPPH at 1 mg/mL (*p* < 0.0001), while the same type of extraction from stressed biomass (*str-N*) exhibited only a 17.79% inhibition of DPPH at 1 mg/mL (*p* < 0.05). When aqueous extracts were used, *std-N* did not exhibit a relevant inhibition of DPPH at any of the concentrations tested, while up to 19.36% inhibition of DPPH was observed with 1 mg/mL of *str-N* (Table 1). 

## 3. Discussion

Microalgae cultured in closed systems such as photobioreactors (PBR) are considered the best option to obtain reliable high-quality biomass production, and high-value metabolites products for pharma applications. The growth strategy adopted is directly related to the final target metabolite to be produced. For example, for *Porphyridium purpureum*, a well-known multi-metabolite producer (phycoerythrin (*Pe*), exopolysaccharides (EPS), and arachidonic acid (AA)), the culture in the PBR must be run in fed-batch mode if the goal is to obtain the maximum amount of the *Pe* pigment. These conditions allow maximum nitrogen concentration within the culture, enhanced phycobiliprotein formation (PbP), and the accumulation of poly-unsaturated fatty acid (PUFA), including AA, in the biomass. In contrast, if the goal is to obtain EPS, the culture must be run in batch mode, in order to stress the culture and reach the stationary phase, allowing the EPS accumulation as a response of the nutrient (mainly nitrogen) depletion [14,30]. Different stress strategies in microalgae have been applied in different microalgae strains to obtain several metabolites from protein, carbohydrate, or lipid fractions [23,31,32,33]. In this study, we evaluated whether stressing (by nutrient depletion) two common commercial marine microalgae cultures resulted in biomass extracts with different bioactivity properties using ovarian SKOV3 cancer cells as a cancer model. 

At present, some studies have simultaneously compared the bioactivity of microalgae extracts obtained under different culture conditions [12,18,19]. For example, Gallardo Rodriguez et al. [19] compared the bioactivity of methanolic extracts from dinoflagellates grown in PBR under different culture modes through the assessment of viability on a neuroblastoma cell line and the anaesthetic effect on Zebrafish. Lauritano et al. [18] published an interesting screening using a wide number of microalgae grown in different culture conditions (at lab-scale only) whose crude extracts were tested for different biological activities. In that study, neither *N. oculata* nor *P. purpureum* were included. Regarding the microalgae strains used in this study (grown at a large scale), there is literature evidencing the bioactivity of *N. oculata* extracts [34,35,36,37] and *P. purpureum* extracts and its secreted polysaccharides [38,39], but none of them test different culture conditions such as nutrient depletion to identify whether the biological effects in cancer cell models are associated with culture conditions.

As a first approach in this comparison, the effects on cancer cell proliferation were evaluated in 2D cultures using the RealTime-Glo™ MT Cell Viability assay and crystal violet staining. The results from the RealTime-Glo assay showed that methanolic extracts of *Nannochloropsis* have antiproliferative activity in SKOV3 cells regardless of the culture conditions. Interestingly, that was not the case for *Porphyridium*. In this case, extracts from standard cultures showed an antiproliferative activity as potent as standard *Nannochloropsis*, but the extracts from the stressed cultures did not reduce cell proliferation. The results obtained from crystal violet staining confirmed the cell viability data obtained by RealTime-Glo. This suggests that under stress culture conditions, such as nutrient depletion, *Nannochloropsis* will keep producing similar levels of the active compound/s responsible for this effect in 2D cultures, whereas *Porphyridium* will alter the amounts of the metabolites that showed bioactivity in 2D cultures. Interestingly, most of the current literature regarding *Porphyridium* extracts mainly refers to the activity of EPS (carbohydrate) fractions [38,39,40,41]. 

One of the aims of this study was also to assess the antitumour activity using 3D spheroid models, which better mimic the tumour microenvironment. To our knowledge, no studies have been published comparing biomass from different culture conditions in 3D models of cancer, and few have started using these models to test biomass from microalgae [42]. *Nannochloropsis* extracts did not reduce the size of the spheroids, although *Porphyridium* extracts from nutrient-stressed conditions, unlike 2D cultures, did show a small reduction. Initially, this result seemed counterintuitive, since 3D models tend to be more resistant to treatments mainly due to the decreased penetration of drugs, areas of quiescent cells, and alterations in gene expression compared to 2D models [26]. When the same type of 3D culture was used to stain live and dead cells present in each spheroid, all the treated spheroids showed a decrease in their live/dead ratios compared to the control. This type of assay is more sensible than size measurement and confirmed the observations from 2D models, revealing that both types of biomass extracts from *Nannochloropsis* also induce cancer cell death in 3D tumour models. Regarding *Porphyridium*, both types of extracts exhibited antitumour activity in this 3D cancer model. This change in antitumour efficacy from the 2D to the 3D model could be explained by a change in the phenotype of cells in the spheroid compared to the phenotype when in the monolayer, as described in [43]. Transition to a different gene expression cell profile from 2D to 3D culture could be sensitising cells to active compound/s contained in nutrient-stressed *Porphyridium* extracts, but absent or at low levels in *Porphyridium* extracts from standard culture conditions. 

When the extracts were tested in the wound-healing assay, *Nannochloropsis* did not show a biologically relevant inhibition of migration. However, extracts from standard cultures of *Porphyridium* did reduce the migration of cells, and this effect was enhanced when extracts from the nutrient-stressed culture were used. Taking these results together with the activity in 2D versus 3D models, it could be suggested that the active metabolite/s enriched in the nutrient-stressed *Porphyridium* are targeting molecules responsible or expressed in processes associated with changes in the cellular morphology and cell–cell interactions, such as reorganisation in 3D or migration [44]. Comparative gene expression studies would help to elucidate which pathways are modulated in these models that could better explain the observed antitumour effects and identify the potential bioactive molecules. As a summary from these cell-based assays, the use of nutrient-depleted biomass extracts from *Porphyridium* exhibited enhanced antitumour effects in SKOV3 cells compared to the biomass extracts from standard conditions, as reflected in the reduced size and viability in 3D models and the reduced migration of treated cells. In the case of *Nannochloropsis*, extracts from both culture strategies provided similar results in the cell models used.

In the literature, it has been reported that groups of molecules with antioxidant activity can also display anticancer activity or sensitise cells to chemotherapy, and these molecules include polyphenolic compounds, carotenoids, sterols, and terpenoids, among others [45]. In the specific case of the strains used in the present study, it has been reported that *Nannochloropsis oculata* contains antioxidant molecules such as sterols that are also able to induce apoptosis in a leukaemia cell line in vitro [36]. In regards to *Porphyridium purpureum*, Juin et al. described that the carotenoid Zeaxanthine, with reported antioxidant properties, also exhibits antitumour activity by promoting apoptosis in a melanoma cell line, as well as sensitising it to the cytotoxic activity of a small-molecule inhibitor (vemurafenib) used in the clinical management of melanoma [46]. For this reason, we found it interesting to perform a preliminary comparison of the antioxidant activity of microalgae extracts from different culture conditions. Apart from this, it is also known that the antioxidant activity of natural compounds can also possess an anticarcinogenic activity (chemoprevention) through modulation of redox status in the cellular environment, preventing DNA damage, mutagenesis, and thus the potential initiation of cancer [47]. One group of these types of molecules are the polyphenolic compounds such as flavonoids. These possess a broad range of pharmacological activities, including antioxidant, antiproliferative, as well as anti-inflammatory, antimicrobial, and estrogenic effects [48,49,50]. As reported in the literature, both strains used in this study are reported to contain polyphenols [51]. For these reasons, we found it interesting to also include a preliminary traditional assessment of antioxidant activity to compare whether different large-scale culture strategies could also affect antioxidant activity, which could also be interpreted as chemo-preventive, chemo-sensitising, or antitumoral. Since the antioxidant activity of *Porphyridium* sp. fractions is widely described in the literature [39,41,52,53], we assessed the antioxidant activity using the DPPH assay only on *Nannochloropsis* biomass extracts as a proof of concept. As reported in the literature, methanolic extracts from standard culture conditions from *Nannochloropsis* exhibited the highest antioxidant activity, whereas aqueous extracts and stressed culture conditions seemed to have a reduced antioxidant capacity [34,37]. These data suggest that in the case of *Nannochloropsis,* the solubility of macromolecules and the nutrient conditions ultimately influences the antioxidant activity of *Nannochloropsis* extracts. Further studies including fractionation of the extracts and chemical evaluations would guide the identification of bioactive compounds from each culture condition that could be playing a role in these activities. 

This study demonstrated how culture conditions can influence the properties of two microalgae strains, including their overall macromolecular composition and biological properties. Such changes in the composition of the biomass have an effect in the bioactivities of these extracts, as described in the 2D and 3D cellular models shown here. More importantly, our data suggest that monitoring of nutrients within the microalgae cultures at a large scale is essential for microalgae biomedical applications as batches with different biological properties will be obtained. In conclusion, microalgae-derived compounds have great potential as natural sources of new anticancer therapies, and innovative strategies to modify and enhance their macromolecule composition will provide new avenues for the development of novel and effective chemotherapeutic agents. 

## 4. Materials and Methods

### 4.1. Microalgae Culture Experimental Design

Both microalgae stains included in this study were acquired from the “Culture Collection of Algae and Protozoa” (CCAP), Scottish Association for Marine Science (SAMS). The target microalgae strains *Nannochloropsis oculata* (CCAP 849/1) and *Porphyridium purpureum* (CCAP 1380/3) were cultured in indoor conditions over several weeks at working volumes of up to 80 L bags. After this period, the algal cultures were grown in photobioreactors in outdoor greenhouse conditions to assess the growth and metabolite production in standard healthy exponential growth conditions and in a stressed stationary condition.

### 4.2. Microalgae Cultivation

The target strains were conditioned in indoor cultures in the Centre for Sustainable Aquatic Research (CSAR), Swansea University (SU), Wales, United Kingdom, prior to scale-up to a higher volume in a greenhouse. Non-axenic cultures of this species were scaled up (using autoclaved seawater for flasks) from 250 mL (A) to a 1 L conical flask (B), then 20 L carboys (C) and finally 80 L (D) bags in indoor conditions using standard F2 commercial media (Cell-hi F2P, Varicon©). From 20 L up, the water pre-treatment method was that used in higher scale cultures. The abiotic parameters in the controlled temperate room to maintain the cultures were a temperature range between 19 and 21 °C, aerated with filtered (0.2 µm) ambient air (0.039% CO_2_) at a rate of 0.6 L (*v/v*) using a 1 mm capillary glass tube inside the flasks/carboys to bubble the air–CO_2_ inside the cultures. The illumination provided was an average of 150 µmol photons·m^–2^ s^−1^ using cool white fluorescent tubes perpendicular to the carboys and with a light:dark cycle of 18:6 h. The use of this photoperiod was chosen due to the similarity to the natural light period in the area (Swansea) during the summer season in Wales. For the experiment run in greenhouse conditions, the target strain cultures from 80 L bags were inoculated in two photobioreactors or Biofences^TM^ (Varicon©) of 800 L working volume each. The Biofences have 48 tubes each (10 m length × 0.032 m diameter each tube) as a light phase (60% of the total volume), and a conical tank as a dark phase (40% of the total volume). The flow rate was set at 200 L/min. Both Biofences have a north–south orientation. Light support was given to the reactor (fluorescence lamps) in order to obtain the same photoperiod regime (18:6 light:dark) found in Wales in the summer season. The seawater (pre-filtered 0.2 µm) used in the trials was disinfected by adding 0.5 mL of sodium chloride per litre of seawater to be treated and left running overnight to reach the same temperature as the inoculum (20 ± 1 °C). The day after, the sodium chloride was neutralised using sodium thiosulphate (0.2 g/L of treated seawater) before the inoculation. The medium used for the microalgae grown in outdoor conditions was the same as the indoor cultures (Cell-hi F2P, Varicon©), and Biofences were supplemented with medium (112 g of Cell-hi F2P) at the beginning of the experiment (Day 0). Pure CO_2_ was injected into both cultures, with an average of ±0.75 L∙h^−1^ during the whole experiment. Samples of biomass and water (supernatant) were taken every other day. For harvesting, a microfiltration unit (Membranology TM, spiral filters 0.2 microns) was used, with a flow rate of 200 L per hour.

### 4.3. Microalgal Analysis

The optical density (OD) method was used to measure the absorbance (abs) or turbidity of the algae culture, and these measurements were carried out in duplicate. A cuvette with 3 mL of natural seawater underwent spectrophotometric analysis (V−1 200 Spectrophotometer, VWR) as a baseline before the sample of algae, and the analysis was carried out at 750 nm, as below this value other pigments can absorb the light, whereas at 750 nm and above they cannot. During the sampling days, at least 50 mL of culture was taken from each Biofence and centrifuged (Beckman Coulter Centrifuge, Avanti J-20XP, JLA rotor) for 10 min at 4000 RPM. The biomass was collected and freeze-dried (ScanVac Cool Safe, LaboGene, Lynge, Denmark) for 48 h for further biochemical analysis. After centrifugation, the supernatant was collected to perform the water chemistry analysis. 

### 4.4. Dry Weight (DW)

To determine the biomass concentration, a known volume of microalgae culture was filtered using a pre-weight dry filter (Whatman filters GF/F, 47 mm diameter, 0.2 microns), washing the biomass 3 times with dH_2_O to remove salt, and then drying the biomass overnight in an oven at 80 °C. The difference between the pre-weight clean filter and the filter with dry biomass yields the biomass concentration of the culture in g/L.

To calculate the DW, the following equation was used:

$$\mathrm{DW}=\frac{\left(Y-X\right)}{V}\times 1000$$

where: *X* = pre-weight of clean filter, *Y* = filter weight after desiccation, and *V* = filtrated culture volume.

### 4.5. Growth Rate (µ) and Duplication Time (DT)

The specific growth rate (*µ*) was determined for all the treatments using the following equation:

μ = (lnN1−lnN0)(T1−T0)

where N0 and N1 are the optical density (OD) at times T0 and T1.

The duplication time (DT) was calculated using the following equation:

DT = (ln(2))μ

### 4.6. Microalgae Biomass Proximal Analysis

#### 4.6.1. Fatty Acids

To measure fatty acids methyl esters (FAMEs), 20 mg of freeze-dried cell biomass was mixed with 3 mL of transesterification solution (methanol/hydrochloric acid/chloroform = 10:1:1, vol/vol/vol) and vortexed for 10 s. Transesterification was conducted at 90 °C for 2 h, and the reaction mixture was cooled down to room temperature. To the mixture, 1 mL of DDI water was added and vortexed. The FAMEs were then extracted by adding 2 mL of extraction solution (hexane/chloroform = 4:1, vol/vol) and centrifuged at 3000 rpm for 3 min for phase separation. Prior to GC analysis, 300 μL of GC-grade hexane was added to resuspend the FAME and the sample was transferred to a GC vial. A Xevo G2S (Waters Corporation, Milford, MA, USA) connected to a 7890B gas chromatograph (Agilent Technologies, Yarnton, UK) was used for the FAME analysis. An injection volume of 1 μL was loaded onto a J&W DB5-MS capillary column (Agilent Technologies, Yarnton, UK) (30 m length × 0.25 mm ID, 0.25 μm film) using helium as a carrier gas. The gas flow rate was 1.2 mL/min and the injector temperature was 280 °C. The oven was programmed as follows: initial temperature 50 °C for 1 min, then ramped at 25 °C per min to 280 °C, which was maintained for 4 min (total run time: 14.2 min). Then, 300 μg/mL (mother stock) of heptadecanoic acid (C17:0) was used as an internal standard, and 10 μL of mother stock was added to each sample. Fatty acids were identified by comparing the obtained retention times with that of known standards (37 component FAME mix, Supelco™) using the software MassLynx (version 4.1) (Waters Corporation, MA, USA).

#### 4.6.2. Proteins

Proteins within the microalgae biomass were extracted using heat (95 °C) and trichloroacetic acid (TCA) [54,55]. The released protein was then quantified through a modified Lowry assay using the reaction between Lowry Reagent D and Folin–Ciocalteu phenol reagent. The absorbance produced at 600 nm was then read spectrophotometrically. Standards of known protein content, BSA (bovine serum albumin), were used to create a standard curve and quantify the protein content in the samples. The time spent at 55 °C varied with the algal species (due to differences in the speed of extraction), and calibration of this step was required for accurate protein quantification of each species.

#### 4.6.3. Carbohydrates

The carbohydrate percentage content in the biomass was calculated by subtracting the protein and lipid percentages obtained from the microalgae samples.

### 4.7. Cell Culture 

The SKOV3 cell line was obtained from The European Collection of Authenticated Cell Cultures (ECACC, Public Health England, Salisbury, UK). Cells were grown in McCoy’s 5a Medium Modified (Gibco), supplemented with 10% heat inactivated foetal bovine serum (FBS, ThermoFisher), 100 units/mL of penicillin, and 100 µg/mL of streptomycin (ThermoFisher). Cells were grown to 80% confluence before passage in complete medium, maintained in a humidified, 5% CO_2_ in-air atmosphere incubator at 37 °C, and the culture medium was changed every 48 h.

### 4.8. Preparation of Microalgae Extracts 

To prepare methanolic extracts, standard and nutrient-stressed dry microalgal biomasses were reconstituted in 100% methanol at 10 mg/mL and sonicated for 30 min at 4 °C in a Bioruptor^®^ Standard sonicator device (Diagenoide). Samples were centrifuged at 10,000 rpm, for 10 min, at 4 °C. Supernatants were recovered, evaporated, and reconstituted in 5% DMSO solution at 10 mg/mL, based on the initial weight of dry biomass used. 

To prepare hydrophilic extracts, standard and stressed dry microalgal biomasses were reconstituted in dH_2_O at 10 mg/mL, sonicated as described above, and centrifuged at 10,000 rpm, for 10 min, at 4 °C. Supernatants were recovered and used only for the antioxidant activity.

### 4.9. Antiproliferative Activity Assays

The effect of methanolic biomass extracts on cell growth was assessed using the RealTime-Glo™ MT Cell Viability Assay (Promega, Southampton, UK) following the manufacturer’s instructions. SKOV3 cells were seeded at 500 cells/well in 96-well tissue culture plates in 100 µL of FBS-stripped medium and cultured in a humidified, 5% CO_2_ in-air atmosphere incubator at 37 °C. After 24 h, cells were treated with control medium or medium containing different concentrations of methanolic extracts (0.0625–1 mg/mL) and RealTime-Glo™ reagents. DMSO was equated in all the concentrations tested. Luminescence, which correlates with cell growth, was measured every 24 h over a 72 h period in a FLUOstar Omega microplate reader (BMG Labtech, Aylesbury Bucks, UK). 

In parallel, after 72 h, the same type of experiment was stained with crystal violet (0.5% *w/v*) solution for 10 min and representative fields for each condition were imaged.

### 4.10. 3D Spheroid Culture and 3D Live/Dead Staining

To grow SKOV3 in 3D spheroids, 1 × 10^3^ SKOV3 cells per well were seeded in 96-well black ULA spheroid plates (Corning) in 100 µL of FBS-stripped medium and cultured in a humidified atmosphere at 5% CO_2_ and 37 °C. After 24 h, cells were organised as spheroids and were treated with vehicle or different concentrations of methanolic extracts. At time 0 and 72 h post-treatment, each spheroid was imaged at 4× magnification with a bright-field inverted microscope. ImageJ software was used to quantify the size of spheroids. The spheroid size was plotted as percentage compared to the respective size of each spheroid at time 0 h.

To evaluate the viability of cells grown in 3D spheroids and using the same culture and treatment conditions as described above, spheroids were stained after 72 h of treatment using a mixture of three stains freshly prepared in PBS, which included 2 µM of Calcein-AM, 3 µM of Ethidium Homodimer−1, and 33 µM of Hoechst 33342 (all from Life Technologies, Carlsbad, CA). This mixture was added very gently to each well and incubated in the dark for 3 h at 37 °C [26]. 

Images were acquired on a Zeiss LSM710 confocal microscope (Carl Zeiss Microscopy, Jena, Germany) using an EC Plan-Neofluor 10×/0.30 Ph1 M27. A stack of 10−14 images separated by <10 µm was acquired, starting at the bottom of the spheroid and covering approximately the lower half of each spheroid.

To analyse the overall intensity of the dead signal in the image and the live signal contained in the spheroid, ImageJ software was used. Z-stacks of each spheroid were transformed to maximum-intensity projections (MIP) and each channel was split into independent images. To analyse the image containing the live signal (Calcein-AM, green), first a mask was created with the image from the bright-field channel, and the mask was used to measure the integrated density of the live fluorescent signal contained in the spheroid. The integrated density of the dead fluorescent signal (Ethidium H−1, red) was also measured on each spheroid MIP image. The data were plotted as the ratio of live versus dead signals.

### 4.11. Migration Assay

SKOV3 cells were seeded at 5 × 10^5^ cells/well in 6-well plates. When the cultures reached 90% confluence, scratches were performed with a 200 µL sterile pipette tip, detached cells were washed with 1× PBS and 2 mL of FBS-free media to reduce proliferation of cells, and they were added to each well together with the specific treatment at a dose with no relevant effect on cell viability (0.0625 mg/mL) to avoid inhibition of closure due to cell death. Images were acquired at 0 and 48 h using a Zeiss inverted microscope at a 4× objective. Reference marks on the bottom of the wells along the scratches were made to align the same fields in each image acquisition time point.

Image analysis of the scratches was performed using the wound-healing size tool, an ImageJ/Fiji^®^ plugin that allows the quantification of the wound area [54,55]. The scratch area was obtained for each field and time point and the percentage of wound closure was calculated using the following formula:

$$\mathrm{Wound}\;\mathrm{closure}\;\%=\;\frac{\left(A_{t\;=\;0}-A_{t\;=\;\Delta t}\right)}{A_{t\;=\;0}}\times 100$$

where “A*_t_*_= 0_” is the area of a specific field at time 0 h and “A*_t_*_= Δ*t*_” is the area of the same field after “*n*” hours of the initial scratch.

### 4.12. DPPH Activity

Antioxidant capacity of biomass extracts was assessed with the DPPH antioxidant assay. One mL of biomass extract, at different concentrations, was mixed with 1 mL of DPPH reagent (0.002% (*w/v*)/methanol solution). After incubation for 30 min in the dark at room temperature, the absorbance was measured at 517 nm using a FLUOstar Omega microplate reader (BMG Labtech, Aylesbury Bucks, UK). A positive control consisting of EPS fraction extracted from another microalgae strain with known antioxidant activity was also included for comparison purposes (data not shown). The results were expressed as percent inhibition of the DPPH radical (% inhibition DPPH), which was calculated according to the following equation, where “Abs_DPPH_” is the absorbance of the DPPH solution without extracts, and “Abs_sample_” is the absorbance of the sample solution:

$$\%\;\mathrm{Inhibition}\;\mathrm{DPPH}\;=\;\frac{\left({\mathrm{Abs}}_{\mathrm{DPPH}}-{\mathrm{Abs}}_{\mathrm{sample}}\right)}{{\mathrm{Abs}}_{\mathrm{DPPH}}}\times 100$$

### 4.13. Statistical Analysis 

Data distributions were assessed for normality using the Kolmogorov–Smirnov test. All data were found to follow a normal distribution, and a parametric test was used for data analysis. An ANOVA test (GraphPad Prism 6, La Jolla, CA, USA) followed by Dunnett’s post hoc test was used to determine significant differences between groups. The test statistic and corresponding *p*-value were reported, and statistical significance was defined as * *p* ≤ 0.05.

## Figures and Tables

**Table 1:** Antioxidant activity of biomass extracts from *Nannochloropsis* measured by the DPPH scavenger assay.

Microalgae Extract (mg/mL)	% Inhibition of DPPH			
	Methanol Extraction		H_2_O Extraction	
	Standard	Stressed	Standard	Stressed
0.0625	2.76	0	7.30	5.40
0.125	4.60	0.92	4.44	7.30
0.25	15.95 *	1.84	0	5.71
0.5	31.60 *	5.21	6.67	10.79 *
1	50.31 *	17.79 *	0	19.36 *

* Denotes significant values, *p* < 0.05.

## Supplementary Materials

The following are available online at https://www.mdpi.com/article/10.3390/md20100627/s1, Figure S1: FTIR from *Nannochloropsis oculata* biomass from two culture conditions (standard and nutrient-stressed).
