# Supplementary material for: Therapeutic Potential of Microalgae-Derived Bioactive Metabolites Is Influenced by Different Large-Scale Culture Strategies
Source: Mar Drugs. 2022 Sep 30;20(10):627. doi: 10.3390/md20100627 (PMC9605503; doi:10.3390/md20100627)
Supplement: Supplementary file 1 [file marinedrugs-20-00627-s001.zip › marinedrugs-1930162-supplementary.pdf]

## Supplementary figure

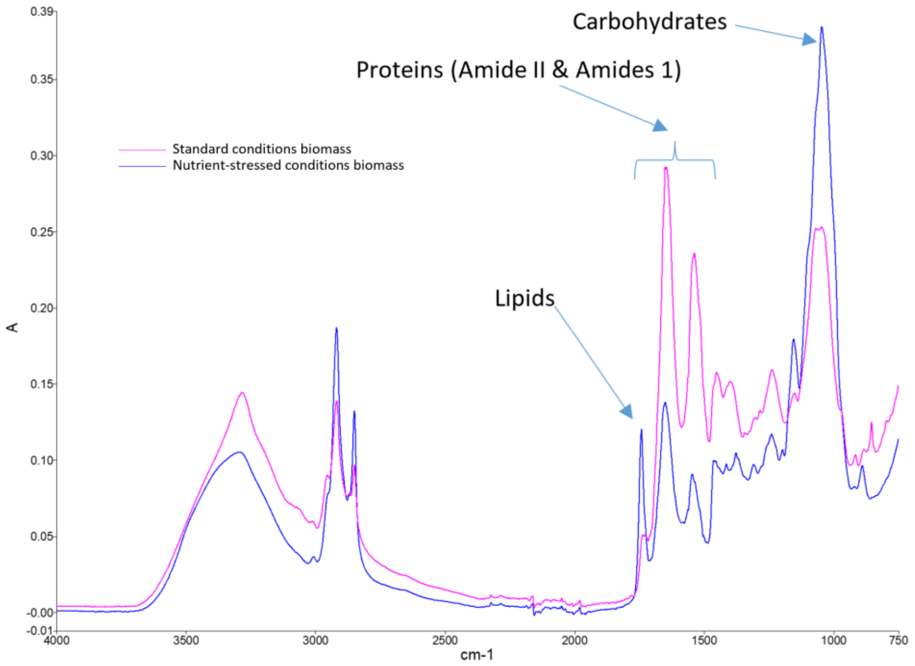


**Figure S1.** FTIR from *Nannochloropsis oculata* biomass from two culture conditions (standard and nutrient-stressed)
